# Laser-assisted hatching improves pregnancy outcomes in frozen-thawed embryo transfer cycles of cleavage-stage embryos: a large retrospective cohort study with propensity score matching

**DOI:** 10.1007/s10815-022-02711-w

**Published:** 2023-01-07

**Authors:** Chaofeng Wei, Shan Xiang, Danqi Liu, Chenggang Wang, Xiaoyu Liang, Haicui Wu, Fang Lian

**Affiliations:** 1 First College of Clinical Medicine, Shandong University of Traditional Chinese Medicine, Jinan, 250000 Shandong China; 2 College of Health, Shandong University of Traditional Chinese Medicine, Jinan, 250000 Shandong China; 3 Integrative Medicine Research Centre of Reproduction and Heredity, The Affiliated Hospital of Shandong University of Traditional Chinese Medicine, Jinan, 250011 Shandong China

**Keywords:** Laser-assisted hatching, Frozen-thawed embryo transfer, Propensity score matching, Live birth

## Abstract

**Introduction:**

Laser-assisted hatching (LAH) is a commonly used adjunct technique; however, its effectiveness has not been fully established.

**Objective:**

We evaluated the effects of LAH on pregnancy outcomes in frozen-thawed embryo transfer (FET) cycles of cleavage-stage embryos.

**Materials and methods:**

This retrospective study involved 5779 FET cycles performed at the Reproductive and Genetic Center in the Affiliated Hospital of Shandong University of Traditional Chinese Medicine between January 2016 and December 2020. After propensity score matching, 3535 FET cycles were included, out of which 1238 were subjected to LAH while the remaining 2297 cycles were non-LAH (NLAH). The primary outcomes were clinical pregnancy rate (CPR) and live birth rate (LBR) while secondary outcomes included implantation rate (IR), biochemical pregnancy rate (BPR), ectopic pregnancy rate (EPR), pregnancy loss rate (PLR), multiple pregnancy rate (MPL), and monozygotic twinning rate (MTR). Logistic regression analysis was conducted to adjust for possible confounders. Subgroup analysis was also performed based on the endometrial preparation regimen.

**Results:**

The LAH group exhibited a higher LBR, compared to the NLAH group (34.9% vs. 31.4%, OR = 1.185, 95% CI = 1.023, 1.374, *P* = 0.024). Additionally, the LAH group showed a decreasing trend in PLR and EPR; however, differences were insignificant (*P* = 0.078, *P* = 0.063 respectively). Differences in IR (24.6% vs. 24.3%), BPR (41.8% vs. 40.4%), CPR (40.7% vs. 38.3%), MPR (14.1% vs. 17.3%), and MTR (1.4% vs. 1.1%) were insignificant. Subgroup analysis revealed that LAH may be more conducive for pregnancy outcomes in hormone replacement cycles.

**Conclusions:**

In summary, LAH has an increased chance of achieving live births. However, further prospective studies should be performed to confirm our findings.

## Introduction

Assisted reproductive technology (ART) has been shown to improve the chances of conception in couples experiencing infertility. Implementation of the two- to three-childbearing policy markedly increased the number of couples wishing to conceive. Although ART has achieved a 30–50% average pregnancy rate [1], the embryo implantation rate (IR) remains low, at about 20–30% [2]. Implantation, an extremely complicated biological process that consists of embryo hatching, localization, attachment, and invasion, is influenced by many factors. Hatching is a pivotal step for successful implantation. During hatching, the embryo gradually gets rid of the zona pellucida (ZP), a phenomenon resembling what happens with insects when they break out of the cocoon. In vitro culture and cryopreservation have been shown to harden or thicken the ZP, resulting in impaired abilities to hatch [3]. As a remedy, an adjunct method referred to as “assisted hatching (AH),” which involves artificial disruption of the ZP, was developed in 1988 [4].

Mechanistically, AH is a technique that manually disrupts the ZP [5]. Lasers, chemical agents, and mechanical methods are often used to achieve ZP destruction, including thinning, drilling, and full-thickness removal [6]. Laser-assisted hatching (LAH) first emerged in the early 1990s [7]. Due to its accuracy, speed, and safety, LAH is the most common method. The use of AH is prevalent in clinical practice, accounting for nearly 44.8% of in vitro fertilization (IVF) cycles in the USA between 2000 and 2010 [8]. According to data from the Society for Assisted Reproductive Technology (SART), 46.7% of frozen-thawed embryo transfer (FET) cycles between 2004 and 2013 underwent AH [9].

Currently, there is no consensus on whether AH improves pregnancy outcomes. An updated Cochrane review indicated that AH can probably increase the clinical pregnancy rate (CPR); however, data quality was not high while the effects of AH on miscarriage rate and live birth rate (LBR) were inconclusive [10]. The first meta-analysis that evaluated the clinical roles of AH in 2003 showed an improved pregnancy rate in poor prognosis patients [11]. However, in 2014, a larger retrospective cohort study reported that AH does not improve pregnancy outcomes, even for patients with poor prognosis [12], findings that were contradicted by a subsequent meta-analysis which reported markedly increased CPR with AH [13]. These differences in outcomes could be due to the high heterogeneity between patients who underwent AH and diversity of the used AH methods. Moreover, there were diverse indications for AH in different ART centers. The most recent guidelines for LAH state that there is insufficient evidence to suggest that LAH is beneficial for patients undergoing FET cycles [14]. In this study, we compared the pregnancy outcomes after day 3 cryopreserved embryo transfer with or without application of the laser zona thinning technique. Propensity score matching (PSM) and multivariate logistic regression analysis were performed to eliminate confounding bias. Our findings elucidate on the significance of AH in ART.

## Materials and methods

### Participants

**Fig. 1:**
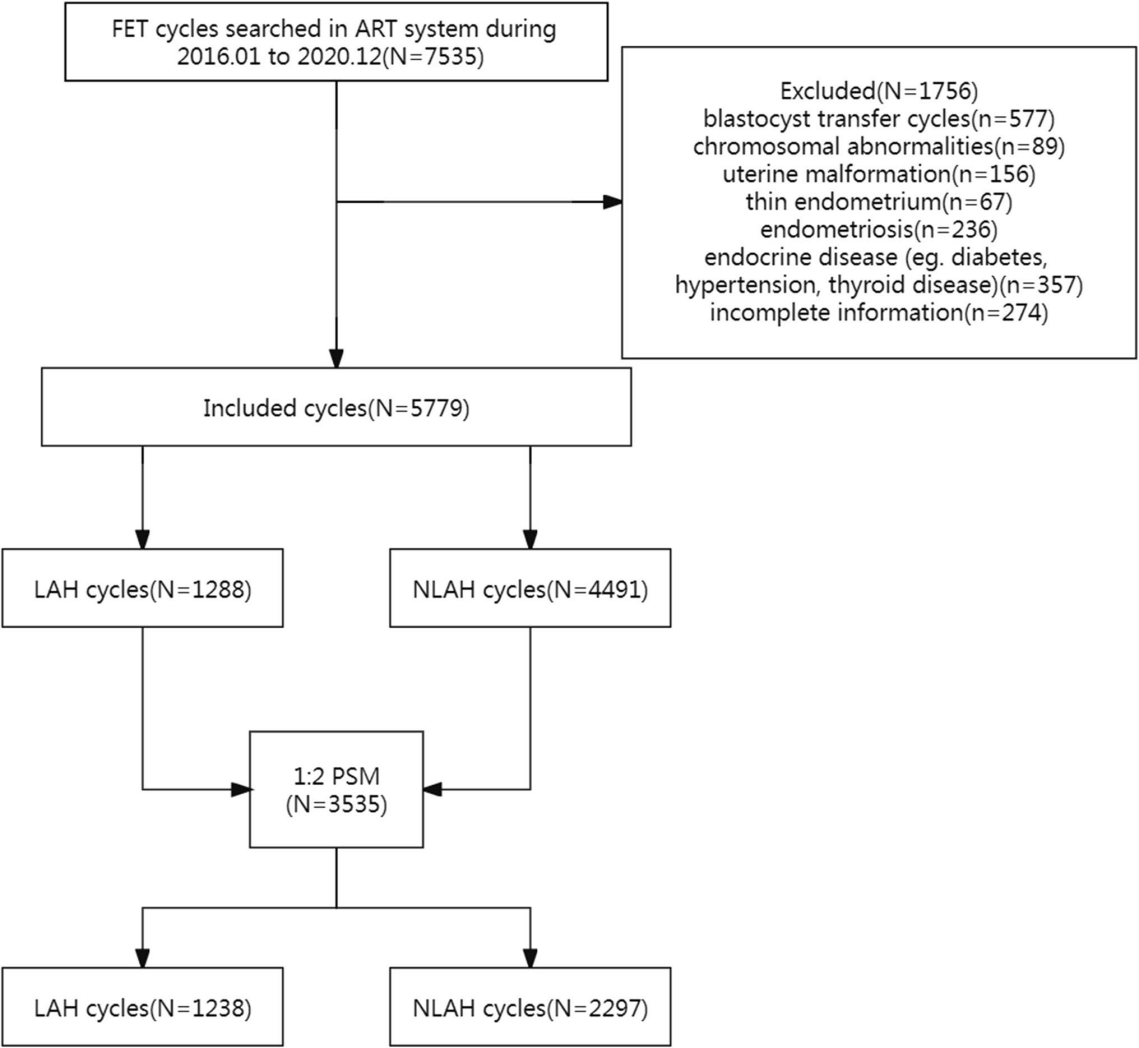
Flowchart showing the selection of study population and matching by propensity score

We retrieved data for 7535 FET cycles in the Reproductive and Genetic Center in the Affiliated Hospital of Shandong University of Traditional Chinese Medicine from January 2016 to December 2020. We included patients with D3 embryo transfer, transplantable embryos after thawing, and who had signed an informed consent prior to receiving LAH. Patients with blastocyst transfer (*n* = 577), chromosomal abnormalities (*n* = 89), thin endometrium (*n* = 67), uterine malformation (*n* = 156), endometriosis (*n* = 236), endocrine disease (such as diabetes, hypertension, and thyroid disease) (*n* = 357), and those with incomplete information on primary outcomes (*n* = 274) were excluded. The included cycles were categorized into LAH and non-LAH (NLAH) groups. The inclusion and exclusion procedures are shown in Fig. 1. The reproductive medicine ethics committee of the Affiliated Hospital of Shandong University of Traditional Chinese Medicine approved this study (no. SZ202109001).

### Endometrial preparation regimen

Hormone replacement therapy (HRT) cycles are appropriate for patients with irregular menstrual cycles. Such patients receive estrogen supplementation (constant or incremental) on the 3rd–5th day of menstruation. In this study, 2000 IU HCG was injected when serum estradiol levels were above 200 pg/mL and endometrial thickness was ≥ 7 mm. The following day, progesterone was administered for endometrial transformation. FET was scheduled on day 4 of progesterone supplementation.

Natural cycles were used for patients with regular ovulation. Follicle and endometrium developments were monitored via vaginal ultrasound from days 10–12 following the onset of menstruation. When ultrasound confirmed the presence of mature follicles and adequate endometrium, HCG (4000 IU) or GnRH-a (0.2 mg) was administered. Progesterone supplementation was initiated 2 days later while FET was performed on day 3 of progesterone supplementation.

Mild stimulation cycles are suitable for individuals with mild ovulation disorders who wish to create an implantation environment by simulating a natural cycle. Ovulation-stimulating drugs were used from days 3–5 of menstruation to stimulate monofollicular growth and promote endometrial growth. Ultrasound monitoring was performed until follicular maturation and endometrial thickness of ≥ 7 mm were achieved, at which time HCG or GnRH-a was injected to trigger ovulation. Transfer was performed 3 days after ovulation.

### Vitrification and thawing

Vitrification was achieved using a vitrification kit (VT101, Kitazato Corporation) as instructed by the manufacturer. Briefly, contracted embryos were placed into the equilibration solution (ES) for 12–15 min for volume recovery. Then, they were transferred to a vitrification solution (VS) for 45–60 s to allow the extracellular fluid of the embryo to be completely replaced with VS. Finally, embryos were loaded at the anterior end of Cryotop with extremely small amounts of VS and immediately transferred into liquid nitrogen.

Vitrified embryos were thawed using a thaw kit (VT102, Kitazato Corporation). Briefly, the anterior end of the Cryotop loaded with embryos was placed into the thawing solution (TS) within 1 s and left to stand for 1 min. Embryos were transferred to the bottom of the diluent solution (DS) for 3 min and washed with washing solution 1 (WS1) for 5 min. Embryos were placed on the surface of washing solution 2 (WS2) and allowed to sink to the bottom (2 times back and forth) for 5 min. Finally, they were moved into the culture medium and incubated for at least 3 h.

### Indication and procedures of assisted hatching

In the absence of guidelines on whether to perform LAH, in our center, the embryologist makes a recommendation when any of the following scenarios are present: (i) female age ≥ 35 years; (ii) ZP thickness ≥ 15 μm; (iii) a history of repeated implantation failure (≥ 2 times); and (iv) multiple sperm still clinging to the ZP when an IVF-fertilized embryo progresses to the cleavage stage. Final decisions are made after full discussions with the couple.

**Fig. 2:**
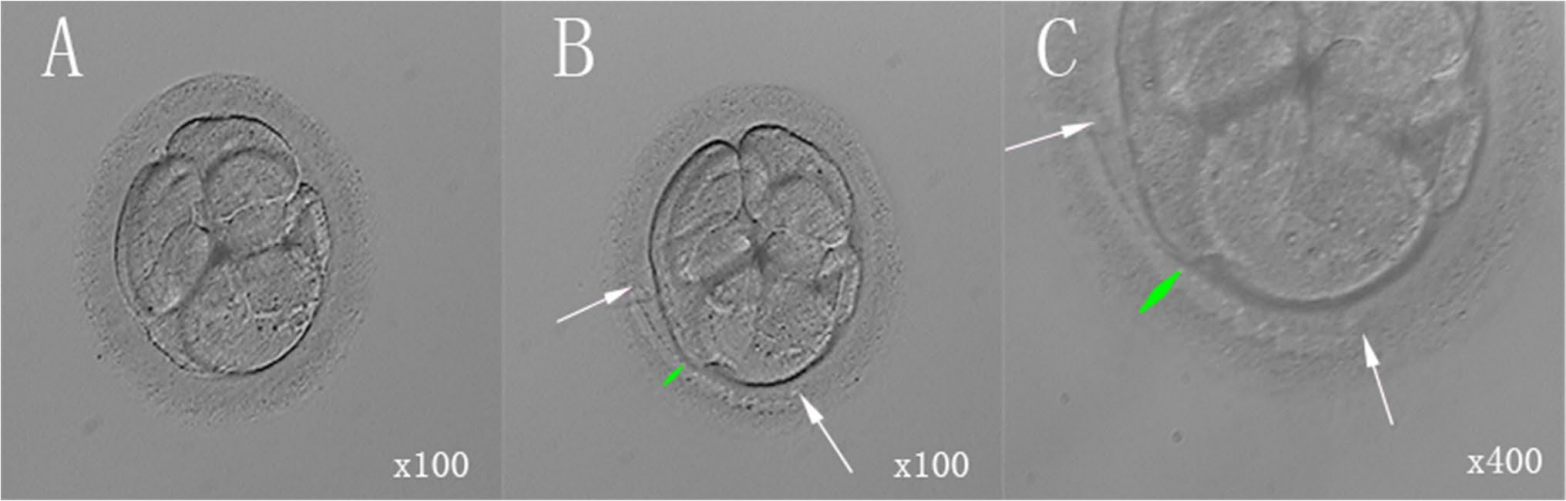
The laser zona thinning procedure for a cleavage-stage embryo. **A** An intact cleavage-stage embryo (× 100 magnification); **B** the 1/4 circumference (the regions between the two white arrows) and the 2/3 thickness (represented by the green double arrow) of zona pellucida (× 100 magnification); **C** the local enlarged image of **B** (× 400 magnification)

Embryos were thawed on the morning of transfer, and laser zona thinning used in the LAH group. Briefly, thawed embryos were placed on a hot plate and observed under an inverted microscope (Nikon, Kanagawa, Japan). Then, the lens was adjusted to the laser position, that is, the site of maximum gap between the embryo and ZP. Proper laser intensity, the quarter circumference of ZP, and the 2/3 thickness of ZP were selected to implement zona thinning. The heat source was always kept away from the blastomeres. After operation, the culture was continued at 37 °C, 6% CO_2_, 5% O_2_, and saturated humidity conditions until embryo transfer. General laser zona thinning procedure is shown in Fig. 2.

### Observation indicators and outcome measures

We analyzed the basic data of patients, which included age, body mass index (BMI), infertility duration, infertility type, cause of infertility, basal follicle-stimulating hormone (bFSH) and basal luteinizing hormone (bLH) levels, number of implant failures, number of embryos transferred, number of good-quality embryos, and endometrial preparation regimens. Embryo grading was performed on day 3 according to the Istanbul consensus (Table 1) [15]. Grade I embryos were regarded as good-quality embryos, while grade I to III embryos were defined as transferrable embryos and were cryopreserved.

**Table 1:** The grading criteria for cleavage-stage embryos

Grade	Number of blastomeres	Fragmentation percentage (%)	Symmetry	Multi-nucleation
I	7 ~ 8	< 10	Even	No
II	7 ~ 8	11 ~ 25	Even	No
	7 ~ 9	< 10	Uneven	No
	≥ 9	< 26	Even	No
III	7 ~ 9	26 ~ 35	Uneven	No
	≥ 6	< 35	Uneven	No
IV	< 6	> 35	Uneven	Yes

For pregnancy outcomes, the primary outcomes were CPR and LBR. Secondary outcomes included IR, biochemical pregnancy rate (BPR), ectopic pregnancy rate (EPR), pregnancy loss rate (PLR), multiple pregnancy rate (MPR), and monozygotic twinning rate (MTR). Biochemical pregnancy was defined as a positive β-HCG (human chorionic gonadotropin) result (β-HCG ≥ 25 IU/L) 14 days after embryonic transfer. Clinical pregnancy was defined as at least one intrauterine gestational sac with heartbeat after 4–6 weeks of embryo transfer. The IR was defined as the number of intrauterine gestational sacs divided by the number of transferred embryos. Pregnancy loss was defined as the occurrence of any abortions during pregnancy, with PLR = (number of abortion cycles/number of all clinical pregnancy cycles) × 100%. Ectopic pregnancy was defined as a pregnancy that occurred outside the uterine cavity. Live birth was defined as delivery of at least one live-born baby per embryo transfer cycle.

### Statistical analysis

Statistical analyses were performed using SPSS version 22.0. Since there was only a small number of missing data for bFSH and bLH (< 10%), we replaced each missing value with mean values of the non-missing item, respectively. Continuous variables were first tested for normal distribution using a histogram and Shapiro–Wilk test. Data that conformed to normal or approximate normal distributions are presented as mean ± SD and were analyzed using Student’s *t*-test; otherwise, data are presented as medians (25%, 75%) and were analyzed using the Mann–Whitney *U* test. Count data are expressed as frequencies (percentage) and were analyzed by the Pearson chi-square test. *P* ≤ 0.05 was set as the threshold for significance.

To reduce the effects of baseline differences on pregnancy outcomes while fully utilizing the obtained data, 1:2 PSM was performed based on female and male age, BMI, infertility duration, infertility type, cause of infertility, bFSH, bLH, number of implant failures, number of transferred embryos, number of good-quality embryos, and endometrial preparation regimen. First, a multivariate logistic regression model was developed to obtain the propensity score representing the probability of patients allocated to the LAH group. Then, the nearest neighbor matching algorithm without replacement was used with a caliper of 0.01 to create a new database containing only matched cases after PSM. These analyses were performed using SPSS 22.0, R (version 2.15.3) and psmatching 3.04 plug-in. Finally, crude and adjusted odds ratios (ORs) with a 95% confidence interval (95%CI) were calculated using binary logistic regression.

Subgroup analysis was performed according to the endometrial preparation regimen to determine whether the effects differed among regimens.

## Results

**Fig. 3:**
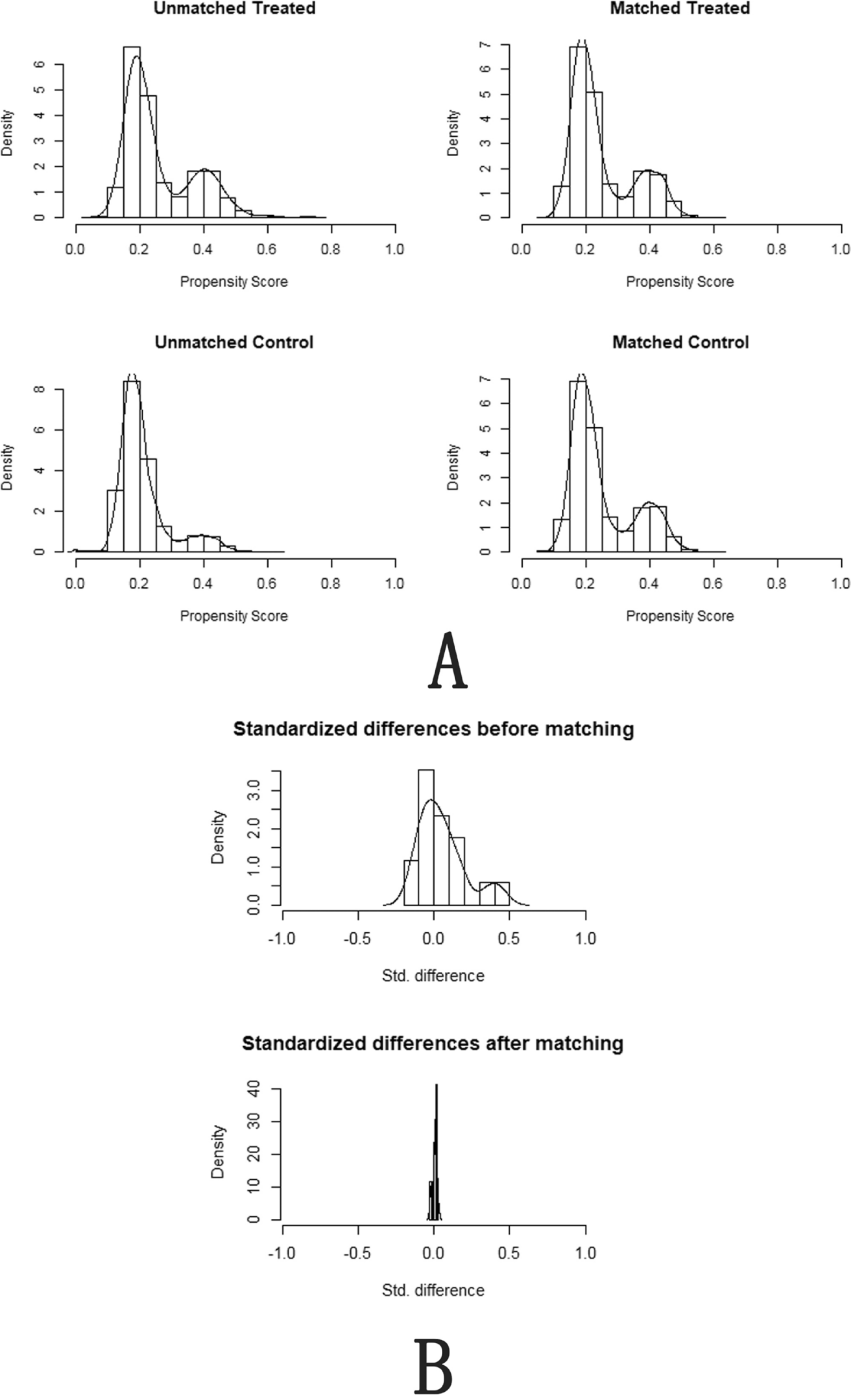
Histograms of propensity score before and after matching

The final analysis included 5779 FET cycles (1288 (22.3%) LAH cycles and 4491 (77.7%) NLAH cycles). After performing the 1:2 PSM, 1238 cycles in the LAH group and 2297 cycles in the NLAH group constituted the matched study cohort (Fig. 1). Graphical assessment of the balance before and after matching is shown in Fig. 3.

### Characteristics before and after PSM

Table 2 shows the characteristics of LAH and NLAH groups before and after PSM. Before matching, there were marked differences across many covariates, including female age (33.74 ± 5.01 vs. 32.82 ± 5.36, *P* < 0.001) and infertility type (proportion of primary infertility: 37.0% vs. 40.1%, *P* = 0.045) among others. After matching, baseline characteristics between the groups were almost well balanced as nearly all differences were minor and non-significant (*P* > 0.05), with a few exceptions (Table 2).

**Table 2:** The basic characteristics of two groups before and after PSM

Characteristics	Before PSM				After PSM			
	LAH group (*N* = 1288)	NLAH group (*N* = 4491)	*t*/*χ*^2^/*z*	*P* value	LAH group (*N* = 1238)	NLAH group (*N* = 2297)	*t*/*χ*^2^/*z*	*P* value
Female age (year)	33.74 ± 5.01	32.82 ± 5.36	5.682	< 0.001*	33.65 ± 4.97	33.50 ± 5.31	0.837	0.403
BMI (kg/m^2^)	23.49 ± 3.56	23.58 ± 3.76	− 0.787	0.431	23.52 ± 3.56	23.45 ± 3.68	0.576	0.565
Infertility duration (year)	3 (2, 4)	3 (2, 4)	− 2.071	0.038*	3 (2, 4)	3 (2, 5)	− 0.822	0.411
Infertility type (%, *n*)			4.033	0.045*			0.616	0.433
Primary infertility	37.0 (476/1288)	40.1 (1799/4491)			36.8 (455/1238)	38.1 (875/2297)		
Secondary infertility	63.0 (812/1288)	59.9 (2692/4491)			63.2 (783/1238)	61.9 (1422/2297)		
Cause of infertility			32.954	< 0.001*			0.738	0.864
Tubal factor	71.3 (918/1288)	69.0 (3099/4491)			71.9 (890/1238)	72.1 (1655/2297)		
Ovulation	13.3 (171/1288)	14.2 (639/4491)			13.7 (169/1238)	14.0 (321/2297)		
Male	4.5 (58/1288)	2.2 (99/4491)			3.5 (43/1238)	3.0 (68/2297)		
Couple	10.9 (141/1288)	14.3 (640/4491)			11.0 (136/1238)	11.0 (253/2297)		
Unknown	0	0.3 (14/4491)			-	-		
bFSH (IU/L)	7.37 (6.10, 8.14)	7.40 (6.18, 7.78)	− 2.445	0.015*	7.37 (6.18, 7.74)	7.40 (6.11, 8.14)	− 2.053	0.040^*^
bLH (IU/L)	5.21 (3.67, 6.43)	5.54 (3.83, 6.45)	− 3.262	0.001*	5.22 (3.69, 6.45)	5.30 (3.70, 6.20)	− 0.775	0.438
Number of implant failures			195.483	< 0.001*			14.101	< 0.001*
≤ 1	72.8 (938/1288)	88.6 (3978/4491)			75.7 (937/1238)	81.1 (1862/2297)		
≥ 2	27.2 (350/1288)	11.4 (513/4491)			24.3 (301/1238)	18.9 (435/2297)		
Number of embryos transferred (*n*)	2 (2, 2)	2 (2, 2)	− 0.509	0.611	2 (2, 2)	2 (2, 2)	− 0.639	0.523
Number of good-quality embryos (*n*)	1 (0, 1)	1 (0, 1)	− 4.084	< 0.001*	1 (0, 1)	1 (0, 1)	− 0.550	0.583
Endometrial preparation regimen (%, *n*)			3.813	0.149			0.194	0.908
Natural cycle	37.8 (487/1288)	36.6 (1644/4491)			37.9 (469/1238)	37.1 (853/2297)		
Replacement cycle	51.0 (657/1288)	53.7 (2410/4491)			51.0 (631/1238)	51.5 (1184/2297)		
Mild stimulation cycle	11.2 (144/1288)	9.7 (437/4491)			11.1 (138/1238)	11.3 (260/2297)		
Male age (year)	34.51 ± 5.50	33.59 ± 5.85	5.233	< 0.001*	34.40 ± 5.44	34.27 ± 5.96	0.678	0.498

Values are presented as mean ± SD or percentage (number/total). *P < 0.05

Abbreviations: *PSM*, propensity score matching; *LAH*, laser-assisted hatching; *NLAH*, non-laser-assisted hatching; *BMI*, body mass index; *bFSH*, basal follicle-stimulating hormone; *bLH*, basal luteinizing hormone

### Pregnancy outcomes

Before matching, differences in pregnancy outcomes had no clinical meaning between the two groups, due to significant differences in their baseline characteristics. After controlling for baseline differences, there were changes in many group differences (Table 3). The IR, BPR, and CPR were slightly higher in LAH group, compared to the NLAH group; however, differences were insignificant. Surprisingly, LBR was markedly increased in the LAH group, compared to NLAH group (34.9 vs. 31.4, *P* = 0.036). In contrast, the LAH group exhibited a decrease in PLR (*P* = 0.078) and a low EPR when compared to the NLAH group (0.7% vs. 1.4%; *P* = 0.063). Differences in MPR and rates of monozygotic twinning between the groups were insignificant (*P* > 0.05).

Due to unbalanced covariates after PSM, logistics regression was performed to adjust for residual confounding. Table 4 shows the results of crude and adjusted ORs. Our results indicate that LBR was increased by the LAH technique, even after adjustment of the aforementioned confounders (OR = 1.185, 95% CI = 1.023–1.374, *P* = 0.024) (Table 4).

**Table 3:** Pregnancy outcomes before and after PSM

	Before PSM				After PSM			
Outcomes	LAH group (*N* = 1288)	NLAH group (*N* = 4491)	*χ*^2^	*P* value	LAH group (*N* = 1238)	NLAH group (*N* = 2297)	*χ*^2^	*P* value
Implantation rate, IR (%, *n*)	24.3 (593/2436)	25.7 (2188/8516)	1.821	0.177	24.6 (575/2342)	24.3 (1050/4329)	0.073	0.788
Biochemical pregnancy rate, BPR (%, *n*)	41.5 (534/1288)	43.3 (1943/4491)	1.331	0.249	41.8 (517/1238)	40.4 (929/2297)	0.577	0.447
Clinical pregnancy rate, CPR (%, *n*)	40.5 (521/1288)	41.0 (1841/4491)	0.122	0.727	40.7 (504/1238)	38.3 (880/2297)	1.945	0.163
Ectopic pregnancy rate, EPR (%, *n*)	0.7 (9/1288)	1.7 (75/4491)	6.592	0.01*	0.7 (9/1238)	1.4 (33/2297)	3.451	0.063
Pregnancy loss rate, PLR (%, *n*)	14.8 (77/521)	18.4 (338/1841)	3.594	0.058	14.3 (72/504)	18.0 (158/880)	3.113	0.078
Live birth rate, LBR (%, *n*)	34.5 (444/1288)	33.5 (1503/4491)	0.453	0.501	34.9 (432/1238)	31.4 (722/2297)	4.387	0.036^*^
Multiple pregnancy rate, MPR (%, *n*)	13.8 (72/521)	18.8 (347/1841)	7.038	0.008*	14.1 (71/504)	17.3 (152/880)	2.406	0.121
Monozygotic twinning rate, MTR (%, *n*)	1.3 (7/521)	0.9 (16/1841)	0.948	0.330	1.4 (7/504)	1.1 (10/880)	0.168	0.682

Values are presented as percentage (number/total)

^*^Indicates *P* < 0.05

Abbreviations: *PSM*, propensity score matching; *LAH*, laser-assisted hatching; *NLAH*, non-laser-assisted hatching

**Table 4:** Relationship between LAH and pregnancy outcomes in different models

Pregnancy outcome	Group	Unadjusted			Adjusted^a^		
		OR	OR 95%CI	*P* value	OR	OR 95%CI	*P* value
Biochemical pregnancy	NLAH^#^						
	LAH	1.056	0.918–1.215	0.447	1.071	0.929–1.234	0.345
Clinical pregnancy	NLAH^#^						
	LAH	1.106	0.960–1.273	0.163	1.120	0.971–1.292	0.118
Ectopic pregnancy	NLAH^#^						
	LAH	0.502	0.240–1.053	0.068	0.516	0.246–1.084	0.08
Live birth	NLAH^#^						
	LAH	1.169	1.010–1.353	0.036^*^	1.185	1.023–1.374	0.024^*^

^a^Adjusted for basal follicle-stimulating hormone (bFSH) and number of implant failures

^#^Reference

^*^Indicates *P* < 0.05

Abbreviations: *LAH*, laser-assisted hatching; *NLAH*, non-laser-assisted hatching; *CI*, confidential interval

### Exploratory subgroup analysis

Subgroup analyses showed that LAH significantly improved pregnancy outcomes in the HRT subgroup; however, differences in natural and mild stimulation cycle subgroups were insignificant (Table 5).

**Table 5:** Subgroup analysis results based on endometrial preparation regimen

Pregnancy outcome	Natural cycle (*N* = 1322)		Replacement cycle (*N* = 1815)		Mild stimulation cycle (*N* = 398)	
	LAH (*N* = 469)	NLAH (*N* = 853)	LAH (*N* = 631)	NLAH (*N* = 1184)	LAH (*N* = 138)	NLAH (*N* = 260)
Implantation rate, IR (%, *n*)	20.0 (175/874)	21.5 (338/1571)	27.6 (331/1198)	25.7 (581/2260)	25.6 (69/270)	26.7 (133/498)
Biochemical pregnancy rate, BPR (%, *n*)	34.5 (162/469)	35.5 (303/853)	46.1 (291/631)	42.7 (505/1184)	46.4 (64/138)	46.5(121/260)
Clinical pregnancy rate, CPR (%, *n*)	33.3 (156/469)	33.8 (288/853)	45.3 (286/631)^*^	40.5 (479/1184)	44.9 (62/138)	43.5 (113/260)
Ectopic pregnancy rate, EPR (%, *n*)	1.1 (5/469)	1.2 (10/853)	0.3 (2/631)^*^	1.4 (17/1184)	1.4 (2/138)	2.3 (6/260)
Pregnancy loss rate, PLR (%, *n*)	17.3 (27/156)	17.0 (49/288)	11.9 (34/286)^*^	18.8 (90/479)	17.7 (11/62)	16.8 (19/113)
Live birth rate, LBR (%, *n*)	27.5 (129/469)	28.0 (239/853)	39.9 (252/631)^*^	32.9 (389/1184)	37.0 (51/138)	36.2 (94/260)

^*^Statistically significant compared with NLAH group in replacement cycles

Abbreviations: *LAH*, laser-assisted hatching; *NLAH*, non-laser-assisted hatching

## Discussion

In this study, 5779 FET cycles (LAH group = 1288: NLAH group = 4491) met our inclusion criteria. We further performed PSM (1:2), which has been effectively used in many studies, to adjust for the basic characteristics between the groups. After matching, 3535 FET cycles (LAH group = 1238:NLAH group = 2297) were included in our analysis. It was found that LAH could reduce the risk of pregnancy loss and increase the chances of live birth.

In previous studies, LAH improved CPR for FET cycles [16–18], but not for fresh embryo transfer cycles [17, 18]. Moreover, LAH was found to significantly increase CPR for patients older than 37 years, which could be used as a routine strategy in FET cycles for such people [19]. In this study, there was a higher CPR in the LAH group. Theoretically, LAH improves CPR by facilitating embryonic implantation. However, in a previous study, embryonic implantation was found to be unrelated to LAH use [20]. Thus, there is a need to determine whether LAH leads to increased CPR and the possible mechanisms.

A limited number of studies have assessed the association between AH and ectopic pregnancy. A previous retrospective study showed that the AH technique increased the risk of ectopic pregnancy [21]. In contrast, another study suggested that younger patients (< 38 years) with thick ZP had comparable EPR, regardless of whether they received AH or not [22]. We found that LAH is associated with relatively low EPR (*P* > 0.05), compared to patients in the NLAH group. These variations in outcomes maybe attributed to the different study populations. Besides, since the occurrence of ectopic pregnancy after embryonic transfer is associated with a history of pelvic inflammatory disease and previous tubal surgery among other factors, inconsistency of the findings should be further analyzed with fewer confounding factors to elucidate on the exact effects of LAH on ectopic pregnancy.

The rate of pregnancy loss is a critical indicator of the therapeutic efficacy of LAH, due to its direct influence on LBR. Data on effects of LAH on miscarriage rate are insufficient to draw rigorous conclusions [23]. We found that LAH decreased the rate of miscarriages, which are often majorly caused not only by chromosomal abnormalities [24] but also by poor implantation. Abnormalities that are associated with embryonic implantation increase miscarriage risks [25]. We postulated that LAH improves embryonic implantation, thereby reducing miscarriage rates. This can be explained by the fact that LAH facilitates implantation onset because embryos can encounter the endometrium earlier than those with intact ZP. Besides, prior to hatching, the artificial gap formed by LAH might act as a passage for exchange of metabolites and growth factors between the embryo and the endometrium, thereby facilitating implantation [26]. These hypotheses may explain, in part, the decreased ectopic pregnancy rate and miscarriage rate associated with LAH.

The ultimate goal of any ART intervention is to obtain healthy live births, and LAH is no exception. For cleavage-stage frozen embryo transfer cases, a previous retrospective study reported a significantly higher LBR with the use of LAH (*P* < 0.001) [27]. Our study supports the finding of this study. In contrast, many other previous studies diverged from this observation [9, 28]. For instance, a Cochrane review [29] and a meta-analysis [30] showed that AH did not have a significant impact on LBR. However, the two studies were limited by the fact that only 7 studies out of 24 AH studies in the Cochrane review treated the LBR as a statistical metric. Similarly, the ratio in the meta-analysis was 4:12. Thus, in the comparison of LBR, reduction in the sample size is a key determinant in achieving significant findings. Therefore, the conclusion that LAH does not affect LBR could exert an unfavorable effect on future patients.

Alteri et al. [31] also held the same view. Therefore, they designed a multicentric randomized controlled trial involving 700 patients to compare the effects of LAH on LBR. The main subjects of the study were patients who received a transfer of vitrified/warmed blastocyst. That is precisely the category of patients that our present study and many other previous studies involved a relatively low number of. Over the past few years, blastocyst transfer has not been advocated in our center. Even though a previous study did not reveal any significant improvements in pregnancy outcomes with LAH in vitrified-warmed blastocysts [32], the findings by Alteri et al. are promising. In addition, a limited number of studies evaluated AH in fresh embryo transfer. A large retrospective study showed that AH had an adverse effect on LBR in fresh embryo transfer [33].

Due to the scarcity of high-quality randomized controlled trials (RCTs), the optimal endometrial preparation regimen has not been established [34]. Findings from our subgroup analysis suggested that LAH is more advantageous for improving pregnancy outcomes during HRT cycles. We posit the possibility that HRT cycles might help physicians to precisely pinpoint the implantation window. The finding was exploratory because potential confounding variables were not taken into account. More rigorous RCTs should be performed to guide clinical practice.

Our findings indicated that the odds of monozygotic twins were not markedly increased by LAH, in tandem with findings by Liu et al. [35]. However, in previous studies, there was a significant correlation between LAH and the increased rate of monozygotic twins [36, 37]. Thus, clinical implications of LAH should be investigated further.

The strengths of our study are the large sample size and the use of PSM. Based on our results, we postulated that LAH improves LBR by preventing miscarriage, which might provide a novel direction for further studies on LAH. It should, however, be noted that the analysis was based on data from patients in our center; therefore, our results may not be extrapolated to other populations. However, this study is associated with various limitations. First, the lack of records on ZP thickness limited our ability to analyze the correlation between ZP thickness and pregnancy outcomes after LAH. Second, due to the retrospective nature of our study and the potential for increased type-I error (false positive) from multiple comparisons, our findings are just exploratory.

In conclusion, LAH might be an effective technique for improving pregnancy outcomes in FET cycles of cleavage-stage embryos. However, there is a need for more prospective randomized studies with extended follow-up duration to assess the benefits and safety of LAH.

## Data Availability

The datasets analyzed during the current study are available from the corresponding author upon reasonable request.
